# Differential genetic and functional background in inflammatory bowel disease phenotypes of a Greek population: a systems bioinformatics approach

**DOI:** 10.1186/s13099-019-0312-y

**Published:** 2019-06-15

**Authors:** Maria Gazouli, Nikolas Dovrolis, Andre Franke, George M. Spyrou, Leonardo A. Sechi, George Kolios

**Affiliations:** 1 Laboratory of Biology, Medical School, National and Kapodistrian University of Athens, Michalakopoulou 176, 11527 Athens, Greece; 2 Laboratory of Pharmacology, Department of Medicine, Democritus University of Thrace, Xanthi, Greece; 3 Institute of Clinical Molecular Biology, Christian-Albrechts-Universität zu Kiel, Kiel, Germany; 4 Bioinformatics ERA Chair, The Cyprus Institute of Neurology and Genetics, Nicosia, Cyprus; 5 Department of Biomedical Sciences, University of Sassari, Sassari, Italy

**Keywords:** IBD, Crohn’s disease, Ulcerative colitis, GWAS, Network analytics, Systems bioinformatics

## Abstract

**Background:**

Crohn’s disease (CD) and Ulcerative colitis (UC) are the two main entities of inflammatory bowel disease (IBD). Previous works have identified more than 200 risk factors (including loci and signaling pathways) in populations of predominantly European ancestry. Our study was conducted on an extended population-specific cohort of 573 Greek IBD patients (364 CD and 209 UC) and 445 controls.

**Aims:**

To highlight the different genetic and functional background of IBD and its phenotypes, utilizing contemporary systems bioinformatics methodologies.

**Methods:**

Disease-associated SNPs, obtained via our own 89 loci IBD risk GWAS panel, were detected with the whole genome association analysis toolset PLINK. These SNPs were used as input for 2 novel and different pathway analysis methods to detect functional interactions. Specifically, PathwayConnector was used to create complementary networks of interacting pathways whereas; the online database of protein interactions STRING provided protein–protein association networks and their derived pathways. Network analyses metrics were employed to identify proteins with high significance and subsequently to rank the signaling pathways those participate in.

**Results:**

The reported complementary pathway and enriched protein–protein association networks reveal several novel and well-known key players, in the functional background of IBD like Toll-like receptor, TNF, Jak-STAT, PI3K-Akt, T cell receptor, Apoptosis, MAPK and B cell receptor signaling pathways. IBD subphenotypes are found to have distinct genetic and functional profiles which can contribute to their accurate identification and classification. As a secondary result we identify an extended network of diseases with common molecular background to IBD.

**Conclusions:**

IBD’s burden on the quality of life of patients and intricate functional background presents us constantly with new challenges. Our data and methodology provide researchers with new insights to a specific population, but also, to possible differentiation markers of disease classification and progression. This work, not only provides new insights into the interplay among IBD risk variants and their related signaling pathways, elucidates the mechanisms underlying IBD and its clinical sequelae, but also, introduces a generalized bioinformatics-based methodology which can be applied to studies of different disorders.

**Electronic supplementary material:**

The online version of this article (10.1186/s13099-019-0312-y) contains supplementary material, which is available to authorized users.

## Introduction

Crohn’s disease (CD) and ulcerative colitis (UC), are the two major manifestations of what is known as inflammatory bowel disease (IBD). They are chronic conditions characterized by prolonged inflammation of the digestive tract and their exact cause is unknown. However, genetics and problems with the immune system have been associated with IBD. Even if recent specific epidemiological data does not exist for Greece, which is the sample source of this work, it was estimated that 2.5–3 million people in Europe are affected by IBD, with a direct healthcare cost of 4.6–5.6 bn Euros/year [1]. Over the last years, a significant number of trait associated gene variants were identified through genome-wide association studies (GWAS) in diverse populations, which strengthened our understanding of complex diseases such as IBD [2]. Regarding European ancestry populations, approximately 200 genome-wide significant (GWS) IBD susceptibility loci [3] have been identified, however, IBD has been associated with significant geographic and ethnic differences in incidence and prevalence [4].

Generally, since GWAS focus on testing association of disease with individual SNPs over the genome and only top-ranked SNPs with the strongest statistical evidence for association are described, GWAS are underpowered to detect loci which have small marginal effect but rather act jointly or interact with trait variability [4, 5]. Thus, more sophisticated analyses such as network-assisted studies that integrate GWAS results are very promising approaches towards the discovery of functionally related genes including those that have a small marginal effect but rather act jointly in disease susceptibility.

Computational approaches have become standard practice in the last decades for managing and analyzing biological data. Due to the accumulative amount of information biological experiments produced, also known as –omics data, the need arose for powerful computational inquiries and storage. Biological databases had to be developed and specialized tools, each targeting specific data types, had to be developed. Contemporary practices and literature [3, 6–8] are focused on these approaches producing more and more knowledge to be consumed. Systems bioinformatics [9] implementations try to combine all this newfound and/or newly appreciated knowledge into comprehensible interactions and provide insights into the patient-disease complex.

In the present study, we employed a bioinformatics pipeline to integrate IBD GWAS results with experimental and bibliographic data via two different approaches; one that informs on pathway-pathway networks and one that provides protein–protein association (via their respective genes) networks. These allowed us to perform network analysis and clustering, to identify sets of interconnected genes and functional pathways associated with each of the two IBD forms and their phenotypes.

More specifically we use the results of our GWAS study of an extended cohort of 573 Greek IBD patients (364 CD and 209 UC) and 441 controls using 89 single nucleotide polymorphisms (SNPs) that showed moderate or strong association in previous studies [6, 10, 11] to perform various network analyses. The data and analysis of CD samples is novel whereas regarding UC we have employed re-analysis of our previously published data using new contemporary bioinformatics approaches. Our results were combined with pathway interaction, and gene co-expression, co-localization, co-occurrence and fusion data to reveal biologically meaningful processes that underlie the risk of IBD. This work aims to have a two-fold impact: to provide scientists who are in with new information on the pathogenesis of IBD and to propose and highlight new methodologies which can be applied on genetic data of different pathological origins.

## Materials and methods

### Study design

**Fig. 1:**
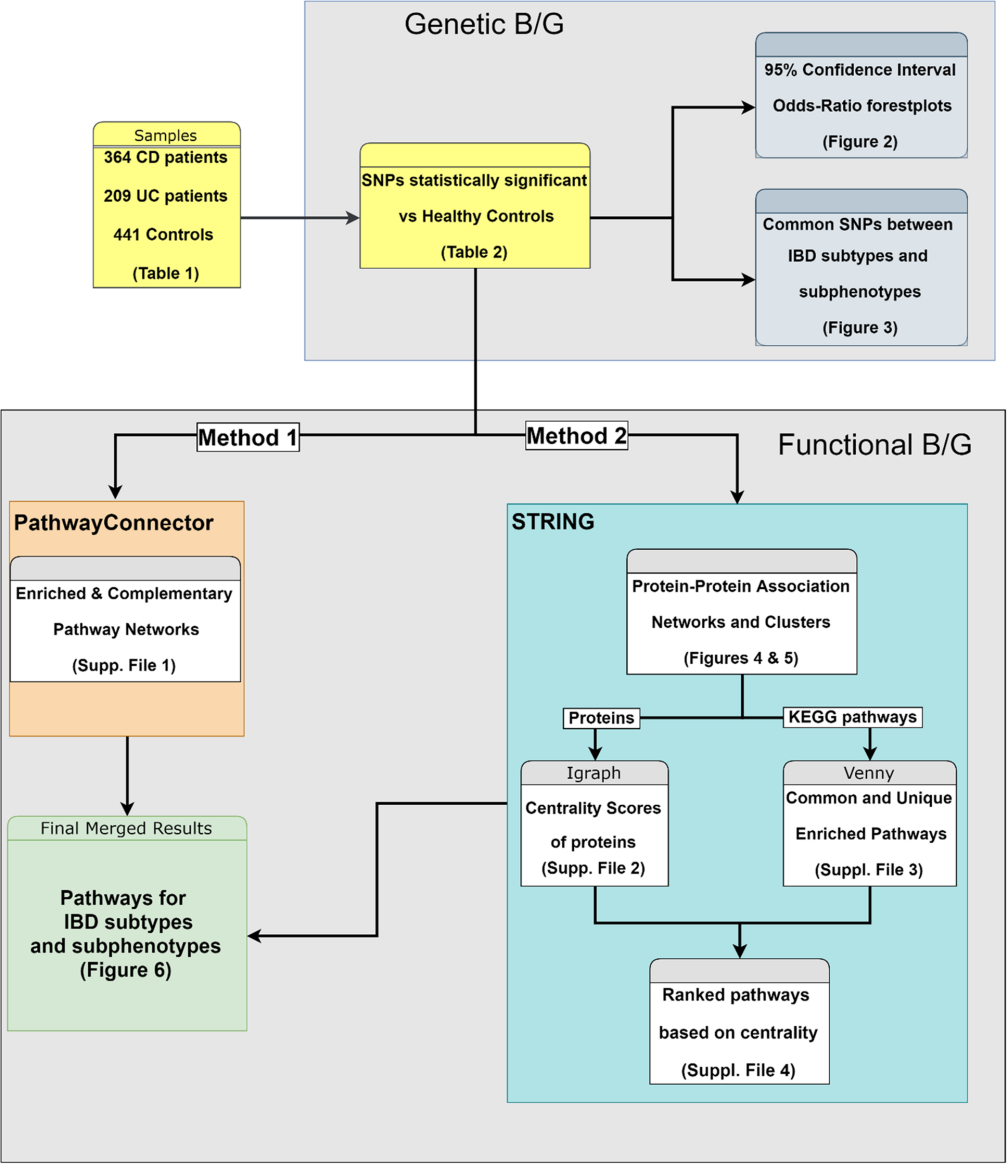
Flow chart showcasing the experimental methodology and study design

The overall experimental design is illustrated as a flowchart in Fig. 1 and will be explained in detail here.

### Samples and DNA isolation

We had conducted GWAS using case–control datasets, totaling 573 Greek IBD cases 364 CD and 209 UC) and 445 healthy controls from unrelated, self-identified Greek individuals as previously described (Table 1) [12]. Our samples were stratified to disease sub-phenotypes according to the Montreal Classification [13] and more specifically CD samples were categorized based on their behavioral subphenotypes (B1: Non-stricturing, Non-penetrating, B2: Stricturing, B3: Penetrating), whereas, UC samples were categorized based on their extent subphenotypes (E1: Ulcerative proctitis, E2: distal UC, E3: pancolitis). None of the patients or controls had a family history of autoimmune disease. The diagnosis of IBD was based on standard clinical, endoscopic, radiological, and histological criteria. Before commencement of the study, the Ethics Committee at the participating centers approved the recruitment protocols. All participants were informed of the study. DNA was isolated from blood with the NucleoSpin blood kit (Macherey–Nagel, Germany).

**Table 1:** Characteristics of case/control sets used

	Crohn’s disease (n = 364)	Ulcerative colitis (n = 209)	Controls (n = 445)
Sex (male/female)	190/174	104/105	233/212
Age (years)			
Range	5–85	15–78	6–85
Mean ± SD	36.21 ± 17.09	44.32 ± 16.88	42.5 ± 15.05
Crohn’s disease location			
Ileal disease	208		
Colonic disease	59		
Ileal and colonic disease	97		
Crohn’s disease behaviour			
Non-stricturing/non-penetrating (B1)	246		
Stricturing (B2)	93		
Penetrating (B3)	25		
Ulcerative colitis disease-extent			
Ulcerative proctitis		20	
Distal UC		124	
Pancolitis		65	

### Genotyping

A genome-wide SNP typing of a discovery panel, using the Affymetrix Genome-Wide Human SNP Array 5.0 was carried out previously at Institute for Clinical Molecular Biology, Christian-Albrechts-University, Kiel, Germany [6, 10]. Part of this panel has been used in previous studies [12].

### SNP quality control and association analysis

The inclusion criteria for the samples in our statistical analysis accounted for SNP missing rate, minor allele frequency and a Hardy–Weinberg Equilibrium exact test p value to rule out genotyping errors. Association analysis was performed on the included samples based on a pairwise comparison of the disease phenotype and sub-phenotypes using a 1 df χ^2^ (Chi square) test. Estimated odds ratios (OR) with a 95% confidence interval (CI) were also calculated for allele 1 (minor) versus allele 2 (major) in our preselected SNPs. Only the SNPs with an asymptomatic p value ≤ 0.05 were considered in our results for further analyses. Quality control and association tests were performed using PLINK [14] v1.90b4.9. The R package metaphor [15] v2.0 was used for the creation of OR plots based on our test results and VENNY [16] was used to identify SNPs common between IBD phenotypes and subphenotypes.

### Signaling pathways enrichment and functional associations

Using the genes carrying the SNPs highlighted by our association analyses, gene-set lists were created as input to the PathwayConnector [17] (Method 1 of the flowchart) and the Search Tool for the Retrieval of Interacting Genes/Proteins (STRING), a database of known and predicted protein–protein associations [18] (Method 2 of the flowchart) platforms.

In Method 1, KEGG [19] was selected as the default signaling pathway database, the top ten Enrichr pathways per set were considered as the initial seed pathways used in the complementary network analysis and edge betweenness was selected as the community detection algorithm for clustering on the complementary pathway network.

For Method 2 each gene of our gene-set was converted to a best matched protein set. The networks were then created using an interaction score of 0.400 (medium confidence) with an enrichment of 30 interactors in total (no more than 20 1st shell and 10 2nd shell interactors), after testing various combinations for the most accurate results based on current knowledge. 1st shell interactors are proteins directly associated with our initial set while 2nd shell ones are those associated with the 1st shell interactors. As active interaction sources all categories had been selected (Textmining: data extracted from the abstracts of scientific literature, Experiments: data extracted from other PPA databases, Databases: data extracted from curated databases, Co-expression: genes that are co-expressed in the same or in other species (transferred by homology), Neighborhood: genes that occur repeatedly in close neighborhood in (prokaryotic) genomes, Gene Fusion: gene fusion events per species, Co-occurrence: proteins linked across species). The Markov Cluster Algorithm (MCL) [20] with an inflation parameter of 3 was applied to the final network for cluster detection based on domain architecture. Edges were created by confidence levels, and disconnected nodes were hidden. Using cytoscape [21], as well as, the igraph [22] and centiserve [23] packages for R, we calculated various network analysis metrics, in order to detect hubs (Degree Centrality), bottlenecks (Betweenness Centrality), shortest path topology (Latora harmonic closeness centrality) and in general nodes (proteins) that play an important role in the protein (PPA) networks. We devised a gene ranking score by using a weighted function, giving Degree centrality a 0.2 factor, Latora Closeness Centrality a 0.3 and Betweenness Centrality a 0.5. This score tries to signify the knowledge represented in literature about the actual significance of those metrics in a protein network [24, 25]. Finally, pathway analysis was performed, on the enriched networks of the disease phenotypes and sub-phenotypes, keeping the KEGG database as reference and the resulting signaling pathway lists were compared using the VENNY online tool to detect and visualize commonalities between them using Venn diagrams. The average combined score of centralities for each protein contributing to a pathway was used to calculate a pathway ranking score.

## Results

As described previously, to elucidate the functional links between single nucleotide polymorphisms (SNPs) and IBD, we used the results from our GWAS analysis to investigate signaling pathways involved in IBD using 2 different computational methods.

**Fig. 2:**
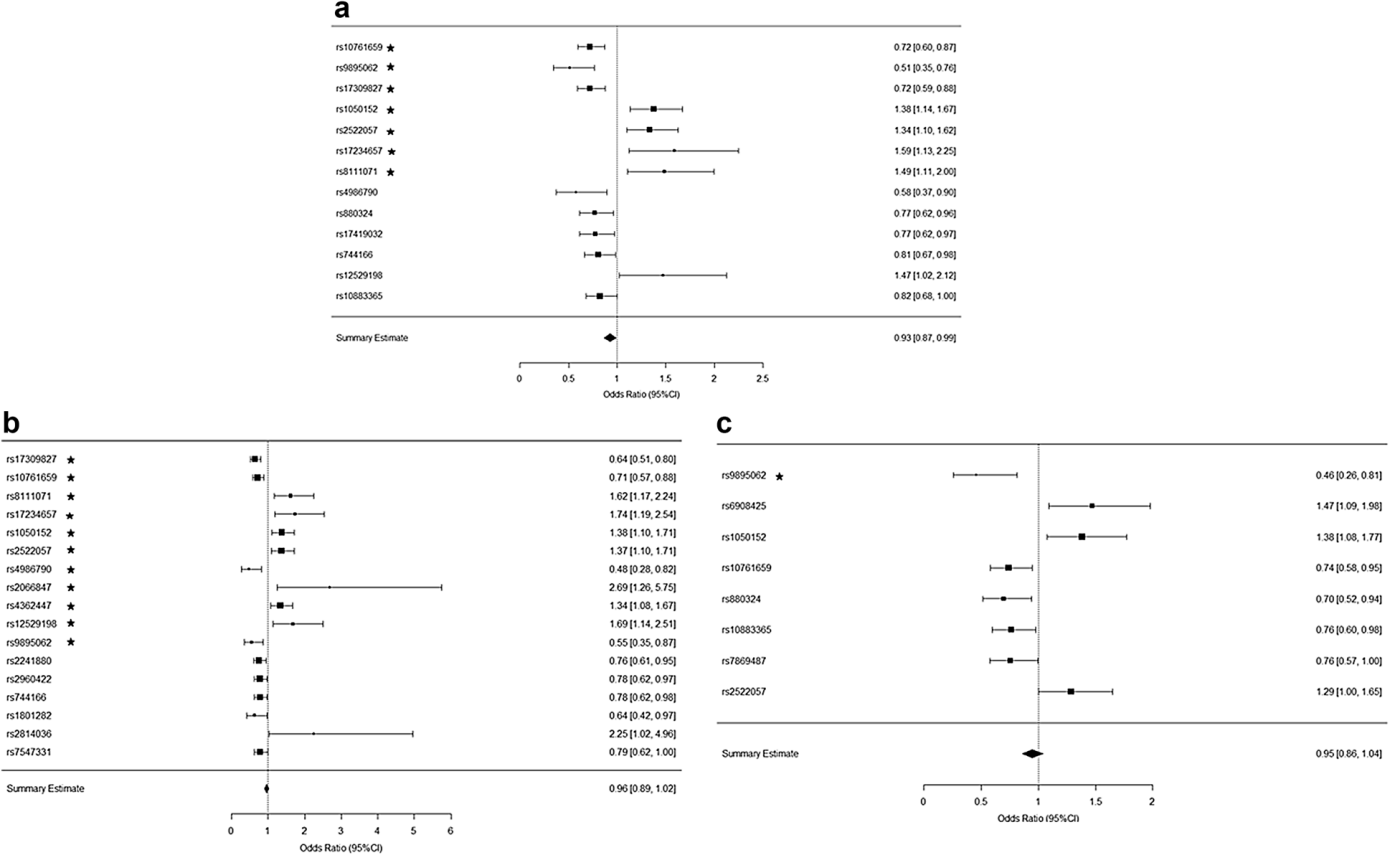
Forest plots of OR ratios for the SNPs highlighted by the SNP analysis performed via plink. These refer to **a** IBD vs HC, **b** CD vs HC, and **c** UC vs HC. All the depicted SNPs statistically significantly relative to the corresponding disease phenotype (*p* value < 0.05 and the ones with the star have a p-value < 0.01). Furthermore, results with an OR score < 1 point to a disease association where the SNP is a homozygote with the minor allele and an OR score > 1 points to a heterozygote

The PLINK analysis results pointed to 17 statistically significant SNPs specific for CD, 8 for UC and 13 generally for IBD compared to healthy individuals (HC), which were used as input in our pathway and enrichment analyses (Table 2). Figure 2a–c showcases the OR diagrams (Forest plots) of these SNPs versus their association to each disease phenotype and sub-phenotype as endoscopically and clinically categorized. The statistical hypothesis here is versus Allele1 and whether the SNP must be a homozygote or heterozygote to be associated with the disease. Results with an OR score < 1 point to a disease association when the SNP is a homozygote and an OR score > 1 points to a heterozygote SNP related to the disease phenotype.

**Table 2:** Overview of the SNPs included in the pathway and enrichment analyses

Phenotype	Locus	Chr.	SNP	A1	F_A (%)	F_U (%)	A2	χ^2^	p-value	OR
IBD	U10	10	rs10761659	C	41.40	49.50	T	11.590	0.000662	0.7212
	STX8	17	rs9895062	A	4.20	7.80	G	11.180	0.000827	0.5136
	C6orf85	6	rs17309827	G	30.50	37.80	T	10.630	0.001115	0.7211
	SLC22A4	5	rs1050152	C	42.00	34.50	T	10.530	0.001172	1.3780
	5025133	5	rs2522057	C	40.50	33.80	G	8.415	0.003721	1.3360
	5p13.1	5	rs17234657	C	9.90	6.40	T	7.038	0.007979	1.5920
	RSHL1	19	rs8111071	A	14.30	10.10	G	7.017	0.008073	1.4880
	TLR4	9	rs4986790	A	3.50	5.90	G	6.140	0.013210	0.5776
	NFATC2	20	rs880324	A	20.00	24.50	G	5.270	0.021700	0.7704
	U1	1	rs17419032	A	19.40	23.80	T	4.760	0.029130	0.7745
	STAT3	17	rs744166	C	34.20	39.20	T	4.629	0.031430	0.8080
	LYRM4	6	rs12529198	A	8.30	5.80	G	4.361	0.036760	1.4730
	NKX2-3	10	rs10883365	A	44.40	49.20	G	3.902	0.048240	0.8248
CD	C6orf85	6	rs1730982	G	27.90	37.80	T	15.090	0.000102	0.6366
	U10	10	rs1076165	C	41.00	49.50	T	9.866	0.001684	0.7086
	RSHL1	19	rs8111071	A	15.30	10.10	G	8.528	0.003498	1.6200
	5p13.1	5	rs1723465	C	10.70	6.40	T	8.391	0.003771	1.7400
	SLC22A4	5	rs1050152	C	42.00	34.50	T	8.090	0.004450	1.3760
	5025133	5	rs2522057	C	41.20	33.80	G	7.692	0.005547	1.3710
	TLR4	9	rs4986790	A	2.90	5.90	G	7.427	0.006427	0.4780
	CARD15	16	rs2066847	–	3.20	1.20	C	7.023	0.008045	2.6890
	U17	17	rs4362447	C	42.20	35.30	T	6.819	0.009019	1.3420
	LYRM4	6	rs1252919	A	9.40	5.80	G	6.813	0.009050	1.6870
	STX8	17	rs9895062	A	4.40	7.80	G	6.786	0.009190	0.5499
	APG16L	2	rs2241880	C	36.10	42.80	T	5.956	0.014660	0.7575
	PPARG	16	rs2960422	A	35.90	41.90	G	4.982	0.025610	0.7768
	STAT3	17	rs744166	C	33.50	39.20	T	4.696	0.030240	0.7803
	PPARG	3	rs1801282	A	5.30	8.10	G	4.396	0.036020	0.6385
	POU2F1	1	rs2814036	A	2.70	1.20	G	4.299	0.038140	2.2540
	ATCL8	1	rs7547331	C	27.00	32.00	T	3.916	0.047840	0.7855
CD B1	C6orf85	6	rs17309827	G	27.80	37.80	T	11.860	0.000572	0.6342
	CARD15	16	rs2066847	–	4.00	1.20	C	10.980	0.000922	3.4740
	LYRM4	6	rs12529198	A	10.90	5.80	G	10.340	0.001305	1.9870
	POU2F1	1	rs2814036	A	3.90	1.20	G	9.246	0.002360	3.2400
	5025133	5	rs2522057	C	42.60	33.80	G	8.519	0.003515	1.4530
	U10	10	rs10761659	C	40.80	49.50	T	8.069	0.004502	0.7044
	SLC22A4	5	rs1050152	C	42.20	34.50	T	6.716	0.009557	1.3860
	TLR4	9	rs4986790	A	2.80	5.90	G	6.228	0.012570	0.4493
	5p13.1	5	rs17234657	C	10.40	6.40	T	5.912	0.015040	1.6860
	RSHL1	19	rs8111071	A	14.90	10.10	G	5.777	0.016240	1.5660
	ATCL8	1	rs7547331	C	25.50	32.00	T	5.005	0.025280	0.7286
	U7	7	rs1558043	C	12.80	17.90	G	4.948	0.026120	0.6744
	NKX2-3	10	rs7081330	A	40.10	33.60	G	4.944	0.026180	1.3220
	U13	13	rs11617463	A	8.90	5.80	C	4.202	0.040370	1.6040
	STX8	17	rs9895062	A	4.70	7.80	G	4.102	0.042830	0.5901
CD B2	APG16L	2	rs2241880	C	32.00	42.80	T	6.000	0.014310	0.6299
	PPARG	3	rs1801282	A	2.90	8.10	G	5.620	0.017760	0.3433
	C6orf85	6	rs17309827	G	28.30	37.80	T	5.008	0.025230	0.6488
	U17	17	rs4362447	C	44.70	35.30	T	4.893	0.026970	1.4860
	5p13.1	5	rs17234657	C	11.40	6.40	T	4.854	0.027580	1.8670
	FLJ44299	16	rs8050910	G	25.70	34.90	T	4.606	0.031860	0.6455
	NKX2-3	10	rs10883365	A	39.60	49.20	G	4.502	0.033860	0.6761
	U10	10	rs10761659	C	40.00	49.50	T	4.302	0.038060	0.6802
	PPARG	16	rs2960422	A	32.90	41.90	G	4.277	0.038630	0.6796
CD B3	RSHL1	19	rs8111071	A	20.50	10.10	G	4.751	0.029270	2.3010
UC	STX8	17	rs9895062	A	3.70	7.80	G	7.447	0.006353	0.4574
	NFATC2	20	rs880324	A	18.40	24.50	G	5.590	0.018060	0.6965
	U10	10	rs10761659	C	42.10	49.50	T	5.826	0.015790	0.7404
	U9	9	rs7869487	C	24.50	30.00	T	3.960	0.046600	0.7558
	NKX2-3	10	rs10883365	A	42.50	49.20	G	4.653	0.031000	0.7624
	5025133	5	rs2522057	C	39.70	33.80	G	3.929	0.047470	1.2870
	SLC22A4	5	rs1050152	C	42.10	34.50	T	6.536	0.010570	1.3820
	CDKAL1	6	rs6908425	C	23.10	17.00	T	6.567	0.010390	1.4720
UC E1	U3	3	rs1462651	C	19.40	9.00	T	8.134	0.004345	2.4400
	5p13.1	5	rs17234657	C	14.30	6.40	T	6.064	0.013800	2.4200
	NCF4	22	rs4821544	C	52.90	38.00	T	5.833	0.015730	1.8340
	DLG5	10	rs1248696	C	0.00	7.40	T	5.538	0.018610	0.0000
	CYLD	16	rs17223195	A	27.00	40.90	G	5.444	0.019640	0.5352
	U13	13	rs11617463	A	12.50	5.80	C	5.087	0.024100	2.3370
	U10	10	rs6601764	C	56.90	44.10	T	4.421	0.035510	1.6790
UC E2	STX8	17	rs9895062	A	2.80	7.80	G	4.551	0.032910	0.3438
	CDKAL1	6	rs6908425	C	23.90	17.00	T	3.894	0.048450	1.5400
UC E3	NKX2-3	10	rs10883365	A	37.90	49.20	G	7.531	0.006065	0.6302
	5p13.1	5	rs1992660	C	28.80	38.30	T	5.832	0.015730	0.6513
	PGLYRP4	1	rs10888557	C	19.80	13.00	G	5.823	0.015820	1.6510
	5025133	5	rs2522057	C	43.10	33.80	G	5.715	0.016820	1.4830
	U10	10	rs10761659	C	40.00	49.50	T	5.546	0.018520	0.6802
	FLJ45139	21	rs2836753	C	47.80	38.40	T	5.448	0.019590	1.4690
	SLC22A4	5	rs1050152	C	43.50	34.50	T	5.249	0.021960	1.4620
	STX8	17	rs9895062	A	3.20	7.80	G	4.997	0.025390	0.3910
	C6orf85	6	rs17309827	G	29.20	37.80	T	4.657	0.030930	0.6787
	5p13.1	5	rs9292777	C	32.10	40.60	T	4.575	0.032440	0.6901
	FAF1	1	rs11205760	C	19.50	26.90	T	4.460	0.034700	0.6567
	FLJ45139	12	rs2836754	C	48.90	40.50	T	4.408	0.035770	1.4070
	NFATC2	20	rs880324	A	17.60	24.50	G	4.126	0.042240	0.6561

The columns from left to right are: phenotype (disease and sub-phenotypes), locus, chromosome, SNP, allele 1 base, frequency of allele 1 in affected individuals, frequency of allele 1 in unaffected individuals, allele 2 base, the score of the basic allelic test χ^2^ (1 df), asymptotic p-value for this test and, estimated odds ratio for allele 1

**Fig. 3:**
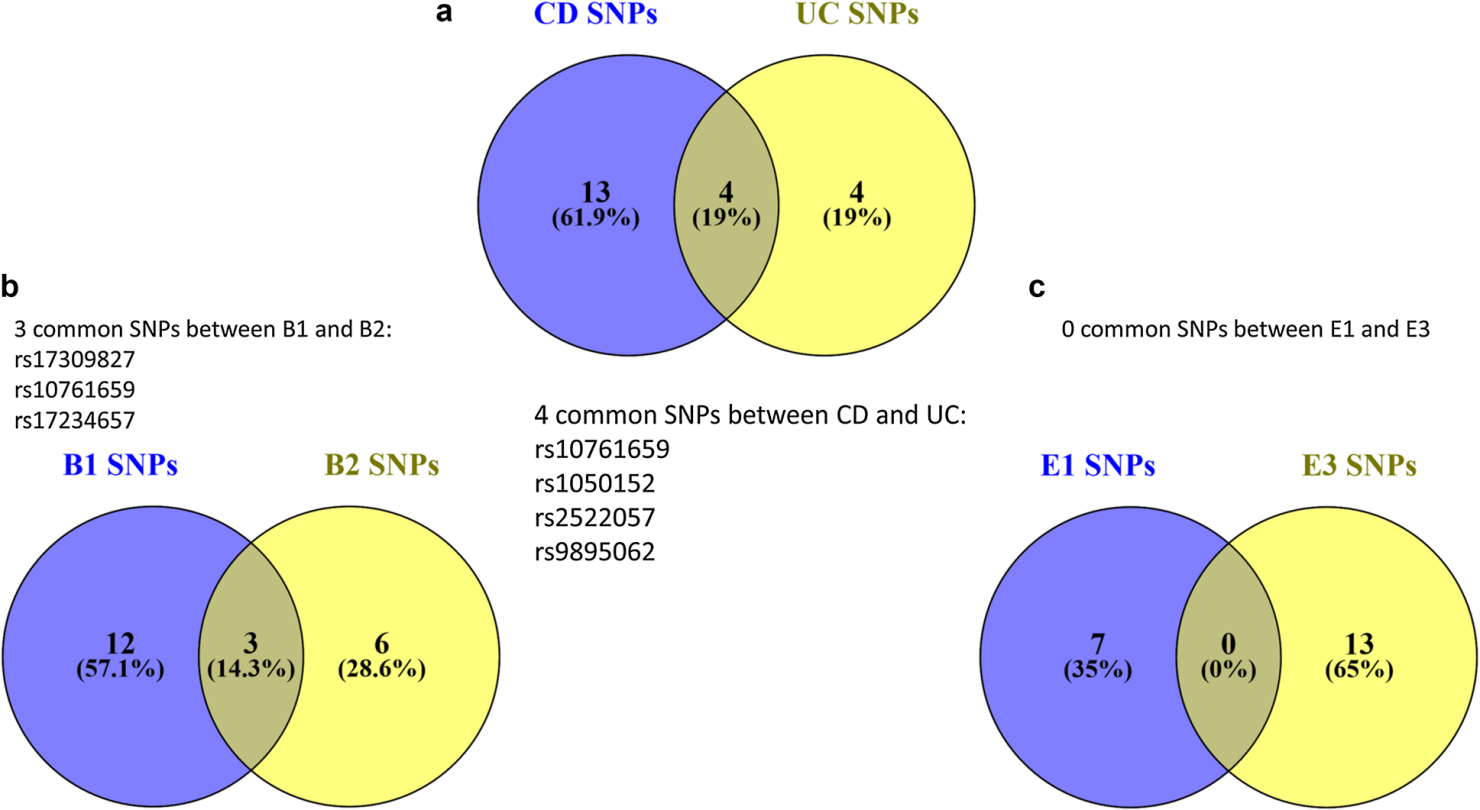
Common SNPs found from the analysis on our datasets, between phenotypes and sub-phenotypes of IBD. **a** 4 common SNPs were found between CD and UC, **b** 3 common SNPs were found between B1 and B2, **c** no common SNPs were found between E1 and E3

Our results revealed regarding CD, 15 SNPs for B1, 9 for B2 and 1 for B3. Concerning UC, 7 SNPs were related to E1, 2 were associated to E2 phenotype and 13 to E3 phenotype (Table 2). It is worth mentioning that the low count of SNPs associated with the B3 and E2 sub-phenotypes is heavily perturbed by the rarity of these cases in our Greek samples and in the worldwide population in general. Figure 3a, in a Venn Diagram, showcases all the SNPs that are common between CD and UC from this initial analysis whereas Fig. 3b the common SNPs between B1 and B2 CD and finally Fig. 3c shows that there are no common SNPs in our results between E1 and E3.

Our results although clearly pointing to a specific and distinct genetic background of the disease phenotypes and sub-phenotypes highlighted the fact that our datasets only contained a handful of genes that don’t allow us to see the bigger picture. It is well known that gene products exert their functions through interactions with other cellular components, and the impact of a genetic perturbation can spread along the links of any functional network the gene product is involved in [26].

To study the role of specific signaling pathways in IBD pathogenesis, we employed Methods 1 and 2 on the gene sets inferred from these SNPs. Genes associated with the B3 and E2 sub-phenotypes gave extremely small datasets to be analyzed so they were disregarded.

Using Method 1 we identified the top 10 pathways after enrichment for all IBD phenotypes and subphenotypes. Moreover, 23 complementary pathways for CD, 11 for UC, 31 for B1, 15 for B2, 24 for E1 and 11 for E3 were detected as interacting with our original 10. The individual results along with visualizations of the complementary networks are included in Additional file 1.

**Fig. 4:**
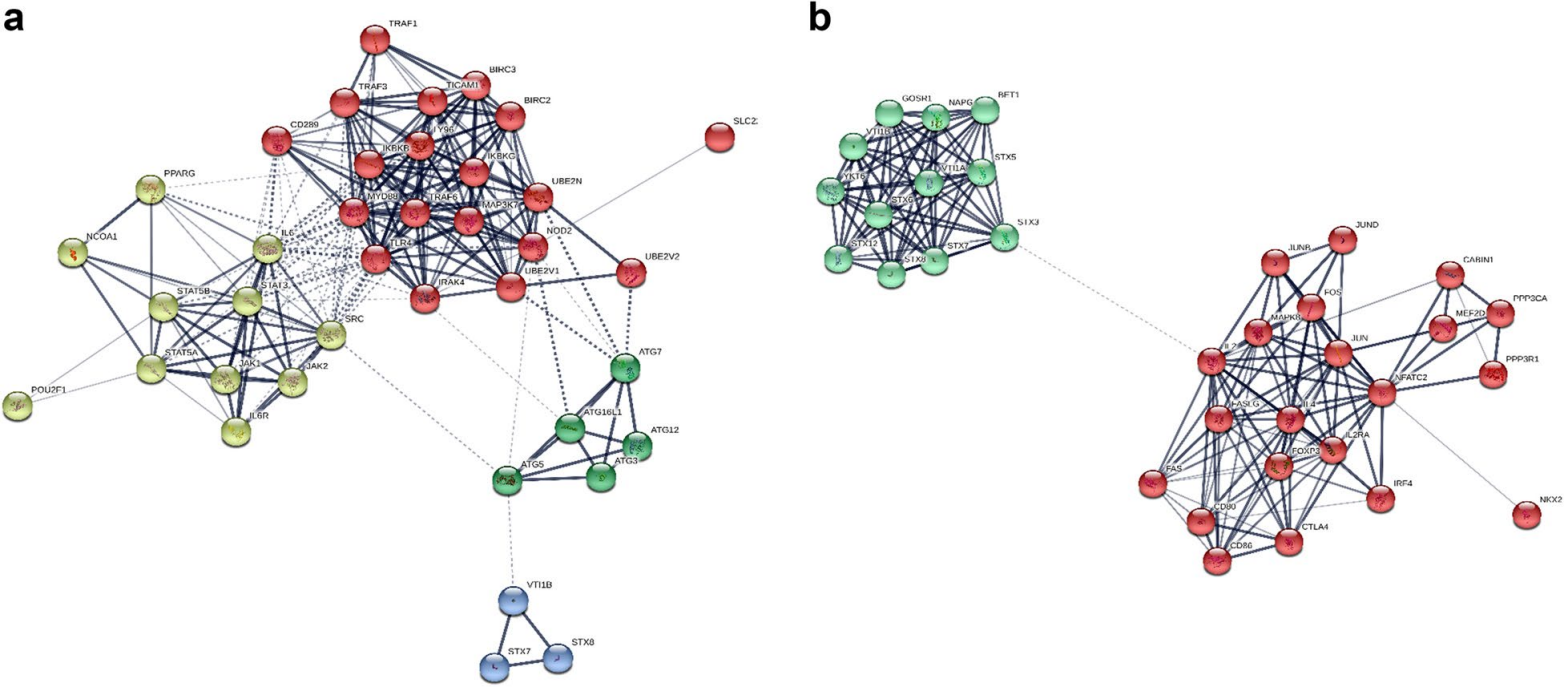
Enriched protein–protein association networks created from the risk genes highlighted from previous analyses for **a** CD and **b** UC. STX7, STX8, VTI1B proteins were found to be common between the 2 networks. 4 distinct clusters detected for CD and 2 for UC

**Fig. 5:**
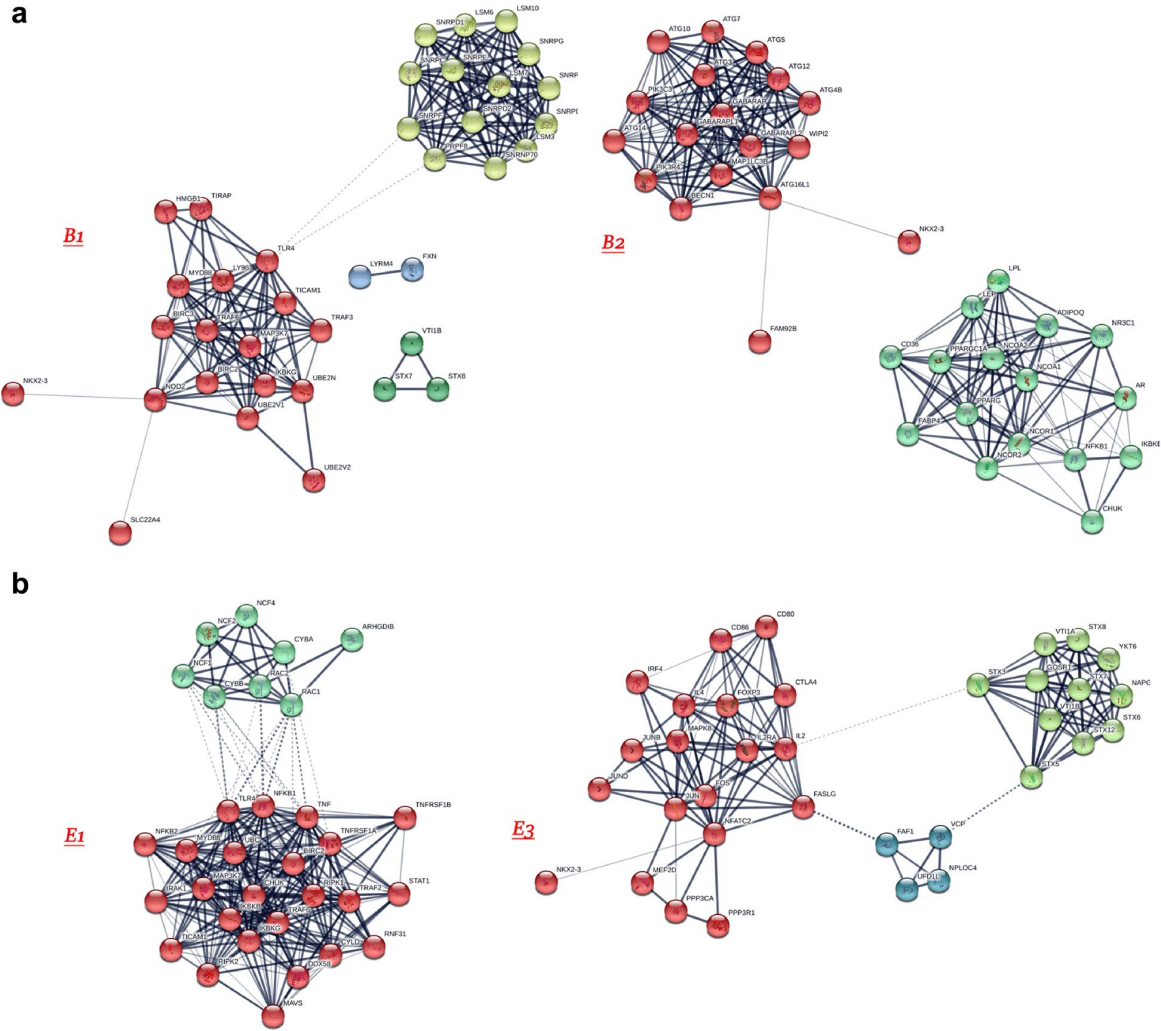
Enriched PPA networks created from the risk genes highlighted from previous analyses for **a** B1 and B2 CD sub-phenotypes and **b** E1 and E3 UC sub-phenotypes. Only the protein NKX2-3 was found to be common between the CD sub-phenotypes, whereas, none were found for UC. 4 clusters were detected for B1, 2 for B2, 2 for E1 and 3 for E3

Using Method 2, we constructed PPA networks and detected signaling pathways. The CD and UC risk genes interaction networks are presented in Fig. 4a, b respectively, whereas Fig. 5a, b showcases the networks created by the B1–B2 and E1–E3 sub-phenotype risk genes as those arose from our previous analyses. Different color groups signify clusters.

The PPA network constructed for CD has 38 nodes, 220 edges and the MCL clustering algorithm has signified 4 clusters, whereas, the UC one has 33 nodes, 164 edges and 2 clusters. In total using the enriched PPA networks only 3 proteins were common between UC and CD: STX7, STX8, VTI1B. The same process for the B1 and B2 CD sub-phenotypes and the E1 and E3 UC sub-phenotypes highlighted: For B1 the enriched PPA network consists of 37 nodes, 187 edges and 4 clusters. For B2 the enriched PPA network consists of 34 nodes, edges and 2 clusters. Only the protein NKX2-3 was found to be common between the 2 enriched networks. The E1 PPA network consists of 32 nodes, 261 edges and 2 clusters, while, the E3 of 34 nodes, 146 edge and 3 clusters. No proteins were found in common between the 2 networks of the UC sub-phenotypes.

Network analysis uses the three different centralities and their subsequent transformation into a combined score has provided, for each phenotype and its sub-phenotypes, a ranked list (Additional file 2) highlighting the proteins most topologically important regarding their protein–protein association networks.

The enrichment process via STRING combined with centrality analysis has also enabled us to study the functional pathways involving the proteins highlighted by the network using KEGG. In total, for the main IBD phenotypes, 26 signaling pathways were found exclusively for CD, 22 for UC and 27 were shared between them. Regarding CD sub-phenotypes B1 and B2, 13 pathways were found exclusively for B1, 21 exclusively for B3 and 15 in common between them. For the UC sub-phenotypes 15 pathways were found exclusively for E1, 30 for E3 and 33 in common between them. Additional file 3 showcases the aforementioned group intersections. Finally, Additional file 4 provides a ranked listing of all the pathways for each phenotype and sub-phenotypes, based on the previous combined scores for each protein, helping identify pathways that might play a significant role to IBD pathogenesis/functional background.

**Fig. 6:**
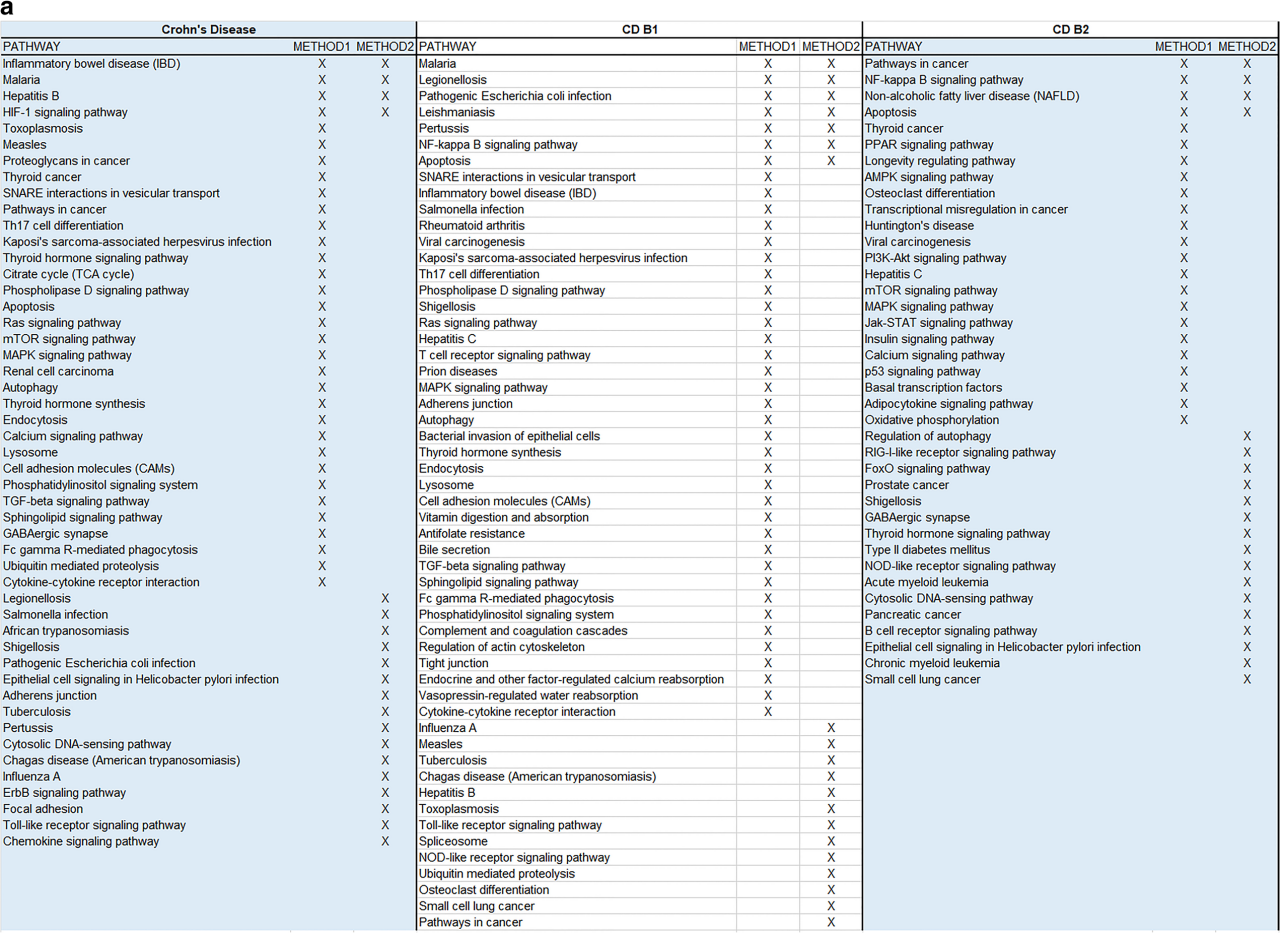

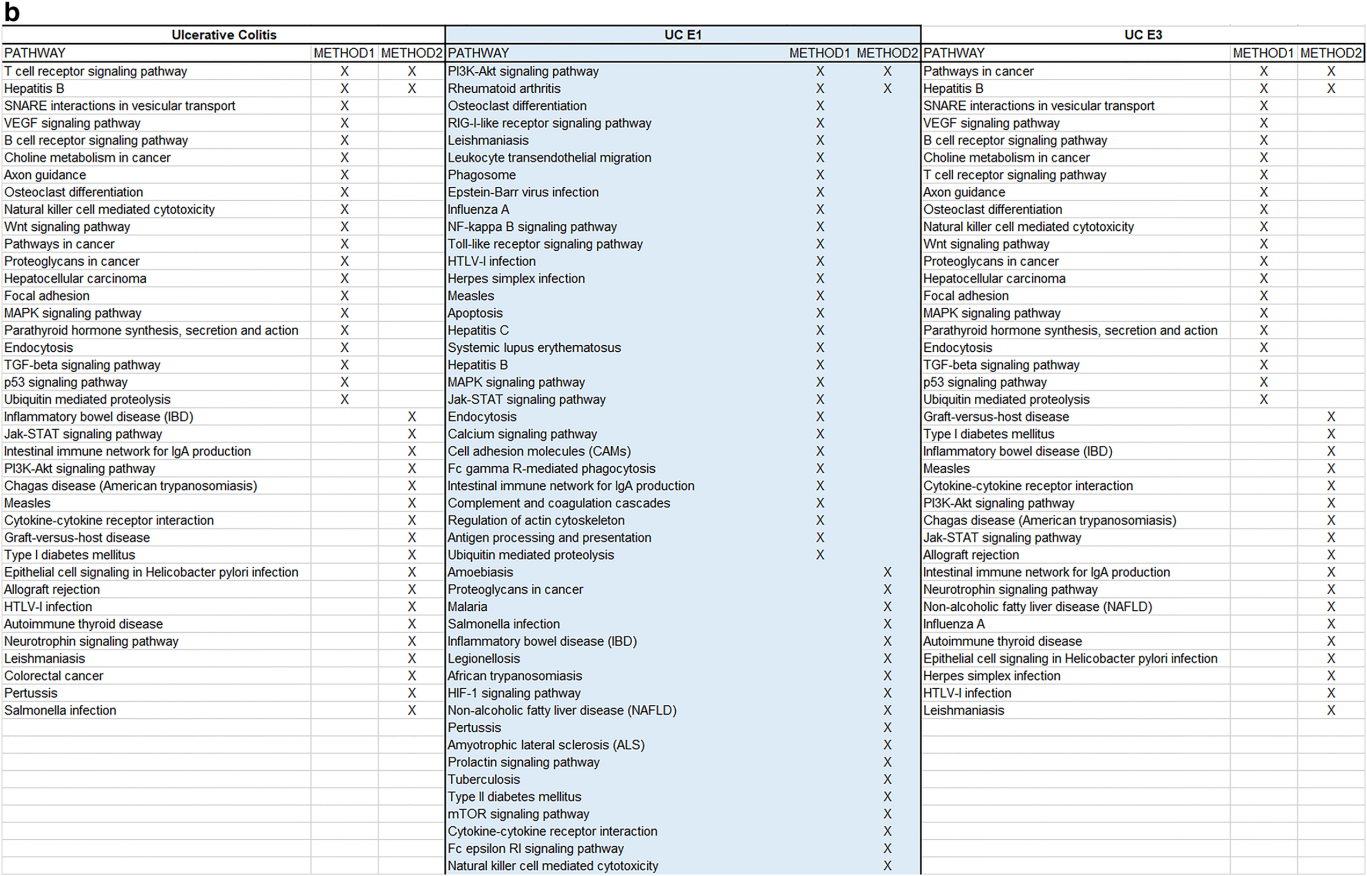
**a** Final merged pathway results from the 2 methods for all CD sub-phenotypes, **b** final merged pathway results from the 2 methods for all UC sub-phenotypes

**Fig. 7:**
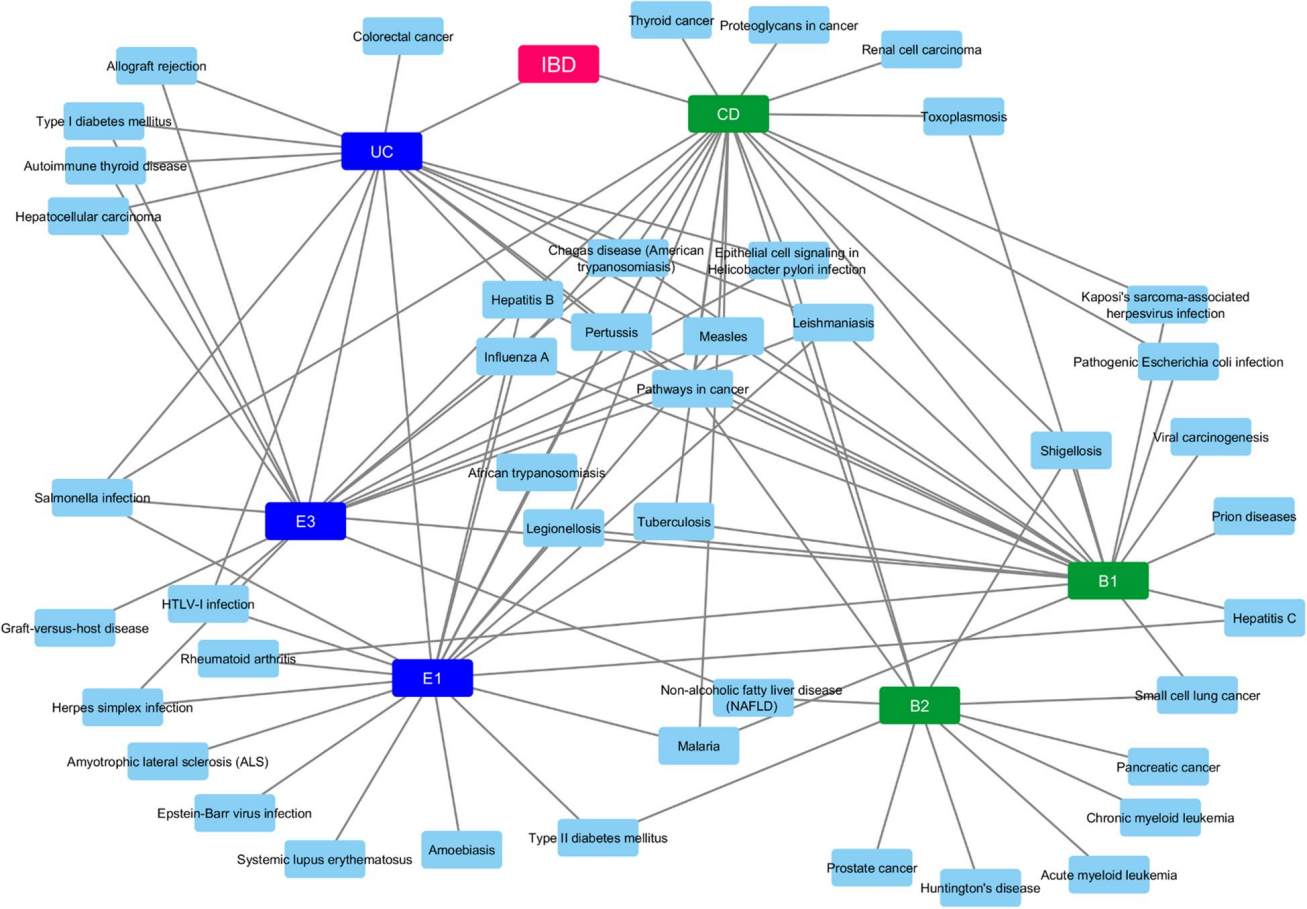
Disease–Disease association network based on molecular background commonalities

To understand better our findings and arrive at a consensus between our methodologies, we have created Fig. 6 which provides common and individually highlighted pathways between Methods 1 and 2 for the IBD phenotypes and subphenotypes. The common ones are four for CD, seven for B1, four for B2, two for UC, two for E1 and two for E3. Finally, using the data from these merged results we constructed a Disease–Disease association network as depicted in Fig. 7. This network allows us to visualize disorders that share molecular mechanisms with our IBD sub-phenotypes.

## Discussion

Recent successes of large GWAS studies have had a large impact on identifying the variants of complex diseases, such as IBD [11, 27–29]. Here, using an integrated pipeline of methodologies we integrate GWAS data of a Greek IBD population with curated databases of fundamental human pathways as well as gene and reaction-based functional networks, in order to obtain novel insights into the potential causal process of IBD and their sub-phenotypes, hopefully leading to specific diagnostic and therapeutic targets.

A novel stride in our present work was the further examination of the main phenotypes of IBD and their sub-phenotypes using a combination of –omics data and network-based approaches. The specificity of the results regarding SNPs, proteins and signaling pathways involved in IBD allows us to shift through general literature findings and pinpoint those that apply exactly to the population under study. We acknowledge that the two approaches showcased in this paper provide us only with a few common results (as depicted in Fig. 6). This is to be expected due to the differences in the methodologies of the two approaches and their intermediate steps. This signifies that when employing various omics methods to extrude conclusions, especially about the functional role of genes, researchers should consider combinational approaches which complement each other, rather than relying on a single method. We also must recognize the limitations of the databases, as highlighted by the KEGG pathway results from both methods, to identify specific disorder pathways when provided with a limited set of genes. Many disorders share common pathophysiological mechanisms like inflammation making it difficult for the database to distinguish the specific disorder under study. This highlights the importance of more specific mechanism-oriented databases.

The use of pathway network connectivity and centrality analysis of the protein–protein association networks, as well as their rankings, not only allows for more unbiased/unmanaged results of important proteins and their role in IBD but also draws attention to specific pathways to be considered out of all those “discovered” by plain pathway analysis methods. By using a weighted approach to combine centralities as shown here, and by modifying the initial scheme presented according to the weight that is desired to be given each time to each centrality, researchers might find the answers to the questions about which nodes are important to a protein association network according to their biological significance/role.

The current analysis implicates a significant number of core pathways indicating an important role among others for IBD, such as Toll-like receptor signaling, TNF signaling, Jak-STAT signaling, PI3K-Akt signaling, T cell receptor signaling, MAPK signaling and B cell receptor signaling pathways components. The NF-kappa B signaling, NOD-like receptor signaling, regulation of autophagy, chemokine signaling, adherents junction pathways were found to be CD specific, whereas the intestinal immune network for IgA production, natural killer cell mediated cytotoxicity, Wnt signaling, cytokine-cytokine receptor interaction, colorectal cancer, VEGF signaling, cGMP-PKG signaling, cell adhesion molecules (CAMs), and Fc epsilon RI signaling pathways seem to be UC specific. When we stratified the cases according to disease sub-phenotypes we identified distinct pathways for the B1 and B2 sub-phenotypes regarding CD, and the E1 and E3 sub-phenotypes regarding UC. Interestingly, the role of most of the identified pathways in IBD pathogenesis and its clinical significance in IBD therapy and diagnostics are well studied [30, 31]. Toll-like receptors are basic mediators of innate host defense in the intestine, involved in maintaining mucosal and commensal homeostasis [32]. Additionally, novel therapies have been developed targeting alternative TNF and ILs signaling (i.e. IL-12/23 axis, IL-6) pathways as well as Jak inhibitors in IBD [33]. It is also well known that combination of disease-associated variants of ATG16L1 and NOD2/CARD15 leads to synergistically increased susceptibility for CD, indicating a possible crosstalk between NOD2- and ATG16L1-mediated processes in the pathogenesis of CD [34]. Notably Kini et al. [35] indicated that changes in signaling through Wnt primarily affected colonic stem cells, whereas Notch affected progenitor function, providing new insights into the development of inflammation and relapse in UC. As depicted in our results, the central role of all these pathways is highlighted.

In the present study the protein–protein association network analysis revealed that 3 proteins were common between UC and CD: STX7, STX8, VTI1B. This is expected since there role of autophagy in the pathogenesis and progression of IBD is well documented [36]. Furthermore, SNARE complexes and their regulators have a key role during inflammation and may present potential therapeutic targets in a wide range of inflammatory diseases such as IBD [37]. SNAREs have recently been implicated in controlling autophagosome development in mammalian cells [38] and the SNAREs vesicle-associated membrane protein (VAMP)7, syntaxin-7 (STX7), syntaxin-8 (STX8), and VTI1B regulate the homotypic fusion of phagophore precursors [39]. These fusion events allow the growth of these structures into a tubular network leading to the formation of phagophores and autophagosomes [40].

Our results further indicated that B1 and B2, CD sub-phenotypes exhibit distinct protein and pathway profiles, and that the NKX2-3 gene was found common in these two entities. These findings are in accordance with previous studies which indicated that NKX2-3 is a susceptibility locus for IBD in Eastern European patients but hasn’t been related to a specific sub-phenotype [41]. However, the B2 network presents two disjointed clusters which might be attributed to the fact that a limited number of SNPs was used in GWAS and the possible links remain outside our initial targets. Regarding UC sub-phenotypes E1 and E3 revealed that they have distinct pathways.

Our observations were also confirmed by the combined centralities network analysis. More specific for CD the proteins identified to have the strongest significant involvement with the disease are TLR4, SRC, NOD2, MYD88 and IL6. These results are not surprising since it is well known that NOD2 is a major genetic risk factor for CD, and NOD2 signal cascade is enhanced by toll-like receptor (TLR) agonists through NF-κB. NOD2 and TLR signaling collaborate to enhance immune responses [42]. TLR4 engages the adaptor MyD88 in combination with the adaptor TIRAP/Mal. Additionally via the signal transduction pathways involving MyD88, IRAK a number of mediators induced that could implicated in the CD pathogenesis such as TNFa, and IL6 [43]. The rest of the proteins identified, are involved in the pathways related to inappropriate immune response to floral components as well as autophagy signaling pathways [44]. Examining the main implicated proteins in CD sub-phenotypes, our results revealed some significant observations. The main proteins related to B1 sub-phenotype are the proteins implicated mainly in TLR and NOD2 signaling pathways (i.e. TLR4, MyD88, NOD2). Regarding NOD2, a previous study suggested that L1007fs mutation, in central Europeans is associated with fibrostenotic disease, [45] but this cannot confirmed in our results and might be be explained by the different ethnic population in our own study. Other proteins correlated mainly with the B1 sub-phenotype are PRPF8, SNRPF as well as TRAF6. Reduced TRAF6 gene expression was found in IBD patients due to hypermethylation [46]. Regarding SNRPF recently Wang et al. [47] identified an antibody against SNRPB, as an autoantibody marker in CD but there are not information related to disease sub-phenotypes. For PRPF8 there are not data available regarding its implication to CD pathogenesis. About the B2 sub-phenotype the autophagy related proteins seem to be more important (ATG12, ATG4B, ATG3 etc.). Even if there are no data supporting the association of autophagy genes with specific CD sub-phenotype, undoubtedly autophagy plays an important role in CD pathogenesis [48]. Conclusively there are distinct protein patterns implicated in these two sub-phenotypes than probably can be used for CD progression prediction.

Interestingly the proteins strongly implicated in UC pathogenesis are distinct from those of CD. IL2, STX3, NFATC2 and JUN seem to have major role in UC. Regarding IL2 it has been shown that Il2^−/−^mice develop IBD most reminiscent of UC [49]. Regarding STX3, a novel mechanism was recently reported, regulating intestinal serotonin transporter (SERT) via PI3K and STX3 [50]. Sikander et al. [51] demonstrated that there may be a potential association between polymorphisms in the (SERT) gene promoter and UC, thus STX3 seems to be important for UC pathogenesis. Considering NFATC2, we know that it is a transcription factor with pleotropic roles [52]. Remarkably, the existing data suggest an important cell-intrinsic role for NFAT family transcription factors in intrinsic negative T cell regulation and Weigmann et al. [53] supported that oxazolone-induced ulcerative colitis and progression to colon cancer are attenuated in NFATC2 KO mice due to ineffective production of IL-6. This suggests that NFATC2 can act as a more generalized modulator of inflammation. Regarding the sub-phenotypes of UC, we observed that E1 is mostly related to proteins such as TLR4, TNF, NFKB1, TNFRSF1A, and others involved in the NF-kappa B signaling pathway. Interestingly E1 sub-phenotype seems to also be strongly associated with Ras-related C3 botulinum toxin substrate 1 (RAC1) protein. It is known that disruption of Rac1 in macrophage and neutrophils of mice protected them against dextran sulphate sodium (DSS)-induced colitis [54]. On the other hand E3 sub-phenotype is mostly related to IL2 protein and also with autophagosomes and inflammation-related proteins i.e. syntaxins and NFATC2 [55, 56]. A strong association for the IL2/IL21 locus with UC is well known [49]. STX3 has a crucial role in trafficking pathways of cytokines in neutrophil granulocytes [57]. Additionally, FASLG seems also to play a basic role in this sub-phenotype and has been documented in the attenuation of apoptosis response to Fas-ligand in active ulcerative colitis [58]. NFATC2 is involved in colitis by controlling mucosal T cell activation in an IL-6-dependent manner and seems to be a potential therapeutic target for UC [56]. Our data indicate that distinct pathways also characterize the UC sub-phenotypes.

Genetic variants and their role in functional changes, though, are not only important in understanding IBD pathophysiology but also understanding treatment-related enigmas like patient response. As previous works [59–63] have shown, traditional IBD treatments like glucosteroids and azathioprine, but also newer approaches like anti-TNF, are all susceptible to inefficiency due to specific genetic polymorphisms. The IBD landscape is vast and includes many factors and pitfalls that should be considered when trying to identify “who” is responsible for disease onset, progression and treatment, by making use of various technical approaches, each targeting a different subsystem [64]. Highlighted among these factors, the microbiome, has become a scientific trend in recent years due to its apparent implication in various diseases, especially IBD. Microbiota dysbiosis appears to either drive or uniquely classify, aspects of IBD like progression [65] and response to treatment [66].

Collectively, our approaches provide important insights into the interplay among IBD risk variants and their related signaling pathways in IBD. All this information is implicated directly to our understanding of the mechanisms underlying IBD and its clinical sequelae. Moreover, by applying these approaches to several disorders and then comparing the results we might be able to understand how key pathophysiological mechanisms can lead to comorbidities previously unknown.

## Additional files

**Additional file 1.** Analysis results via PathwayConnector for all our studied phenotypes except B3 and E2 due to the limited amount of statistically significant genes after the initial GWAS analysis. For each phenotype we report the top 10 statistically significant pathways after enrichment, the newly associated pathways via the construction of a complementary network and finally the network’s visual representation. All the network visualization figures are high resolution and can be saved and viewed individually. (Index: Page 2: Crohn’s Diseaseq Page 3: B1 CD; Page 4: B2 CD; Page 5: Ulcerative Colitis; Page 6: E1 UC; Page 7: E3 UC).

**Additional file 2.** The ranked proteins associated with each IBD phenotype and sub-phenotype after centrality analysis, in their respective sheets.

**Additional file 3.** Unique and shared KEGG pathways between different phenotype groupings after enrichment via STRING: CD vs UC, B1 vs B2 and E1 vs E3. The results are shown in the respective sheets. a) CD vs UC, b) B1 vs B2, c) E1 vs E2.

**Additional file 4.** The table represents all the KEGG pathways per IBD phenotype and sub-phenotype by utilizing the results in Additional files 2 and 3. These have all been ranked using the protein centrality scores for the proteins contributing to each one of them as explained in the manuscript.

## Data Availability

All data and materials are available upon request.
