# Supplementary material for: Differential genetic and functional background in inflammatory bowel disease phenotypes of a Greek population: a systems bioinformatics approach
Source: Gut Pathog. 2019 Jun 15;11:31. doi: 10.1186/s13099-019-0312-y (PMC6570833; doi:10.1186/s13099-019-0312-y)
Supplement: Supplementary file 1 — Additional file 1. Analysis results via PathwayConnector for all our studied phenotypes except B3 and E2 due to the limited amount of statistically significant genes after the initial GWAS analysis. For each phenotype we report the top 10 statistically significant pathways after enrichment, the newly associated pathways via the construction of a complementary network and finally the network’s visual representation. All the network visualization figures are high resolution and can be saved and viewed individually. (Index: Page 2: Crohn’s Diseaseq Page 3: B1 CD; Page 4: B2 CD; Page 5: Ulcerative Colitis; Page 6: E1 UC; Page 7: E3 UC). [file 13099_2019_312_MOESM1_ESM.docx]

This supplementary file contains the analysis results via PathwayConnector for all our studied phenotypes except B3 and E2 due to the limited amount of statistically significant genes after the initial GWAS analysis.

For each phenotype we report the top 10 statistically significant pathways after enrichment, the newly associated pathways via the construction of a complementary network and finally the network’s visual representation.

All the network visualization figures are high resolution and can be saved and viewed individually.

Index

Page 2: Crohn’s Disease

Page 3: B1 CD

Page 4: B2 CD

Page 5: Ulcerative Colitis

Page 6: E1 UC

Page 7: E3 UC

# Crohn’s Disease

**Top 10 statistically significant pathways after enrichment**

| Description | Combined Score |
| --- | --- |
| Inflammatory bowel disease (IBD) | 13.264081 |
| HIF-1 signaling pathway | 10.548006 |
| Toxoplasmosis | 10.389376 |
| Measles | 9.382529 |
| Hepatitis B | 9.403571 |
| Proteoglycans in cancer | 8.461796 |
| Thyroid cancer | 5.583223 |
| SNARE interactions in vesicular transport | 5.119122 |
| Malaria | 5.180126 |
| Pathways in cancer | 6.052924 |

**Newly associated pathways via the complementary network**

Th17 cell differentiation, Kaposi's sarcoma-associated herpesvirus infection, Thyroid hormone signaling pathway, Citrate cycle (TCA cycle), Phospholipase D signaling pathway, Apoptosis, Ras signaling pathway, mTOR signaling pathway, MAPK signaling pathway, Renal cell carcinoma, Autophagy, Thyroid hormone synthesis, Endocytosis, Calcium signaling pathway, Lysosome, Cell adhesion molecules (CAMs), Phosphatidylinositol signaling system, TGF-beta signaling pathway, Sphingolipid signaling pathway, GABAergic synapse, Fc gamma R-mediated phagocytosis, Ubiquitin mediated proteolysis, Cytokine-cytokine receptor interaction

**Visualization of the complementary network (green nodes = newly associated)**


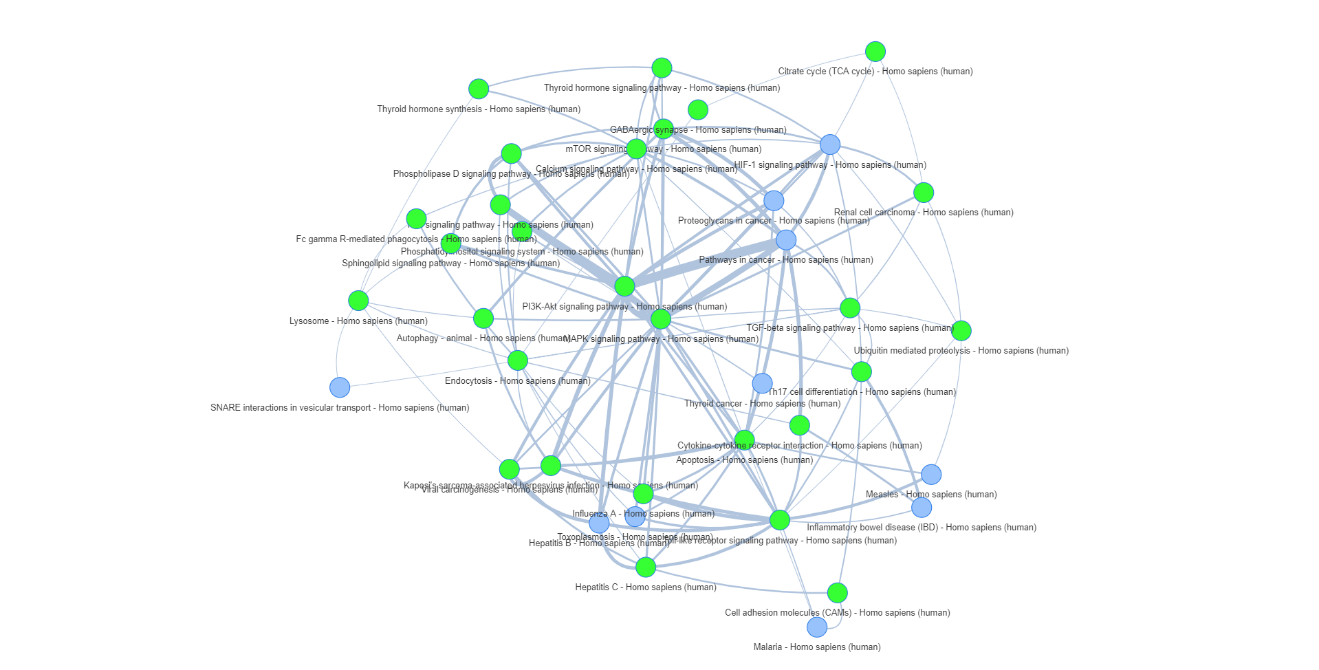


# B1 CD subphenotype

**Top 10 statistically significant pathways after enrichment**

| **Description** | **Combined Score** |
| --- | --- |
| SNARE interactions in vesicular transport | 6.965793 |
| Malaria | 6.363446 |
| Legionellosis | 5.887479 |
| Pathogenic Escherichia coli infection | 5.579274 |
| Inflammatory bowel disease (IBD) | 5.53752 |
| Leishmaniasis | 5.013654 |
| Pertussis | 4.483722 |
| Salmonella infection | 4.201795 |
| Rheumatoid arthritis | 4.214469 |
| NF-kappa B signaling pathway | 4.072872 |

**Newly associated pathways via the complementary network**

Viral carcinogenesis, Kaposi's sarcoma-associated herpesvirus infection, Th17 cell differentiation, Phospholipase D signaling pathway, Shigellosis, Apoptosis, Ras signaling pathway, Hepatitis C, T cell receptor signaling pathway, Prion diseases, MAPK signaling pathway, Adherens junction, Autophagy, Bacterial invasion of epithelial cells, Thyroid hormone synthesis, Endocytosis, Lysosome, Cell adhesion molecules (CAMs), Vitamin digestion and absorption, Antifolate resistance, Bile secretion, TGF-beta signaling pathway, Sphingolipid signaling pathway, Fc gamma R-mediated phagocytosis, Phosphatidylinositol signaling system, Complement and coagulation cascades, Regulation of actin cytoskeleton, Tight junction, Endocrine and other factor-regulated calcium reabsorption, Vasopressin-regulated water reabsorption, Cytokine-cytokine receptor interaction

**Visualization of the complementary network (green nodes = newly associated)**
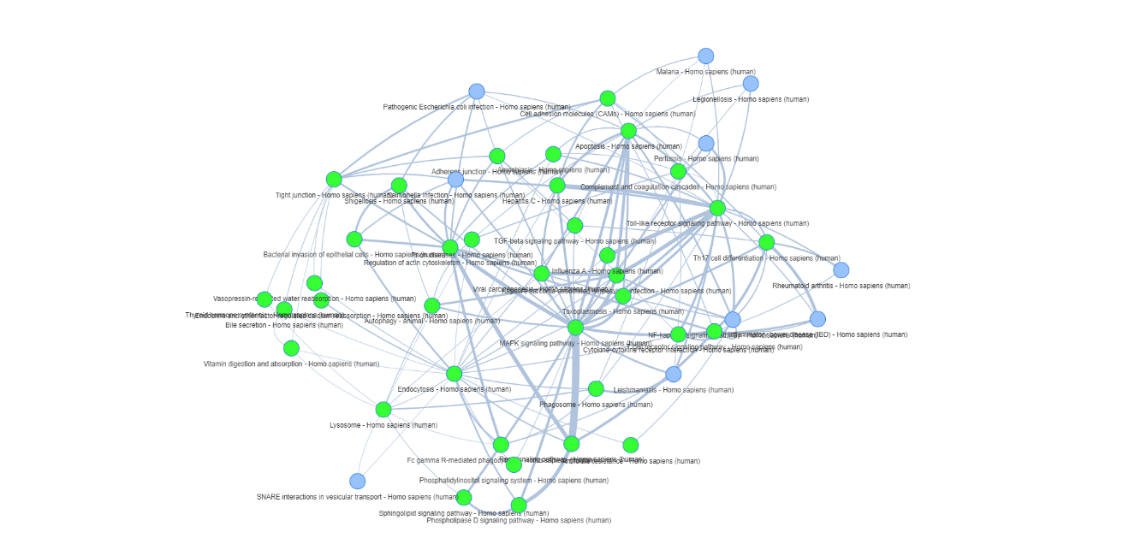


# B2 CD subphenotype

**Top 10 statistically significant pathways after enrichment**

| **Description** | **Combined Score** |
| --- | --- |
| Thyroid cancer | 8.243498 |
| PPAR signaling pathway | 6.106493 |
| Longevity regulating pathway | 6.148715 |
| AMPK signaling pathway | 5.189774 |
| Osteoclast differentiation | 5.218721 |
| Transcriptional misregulation in cancer | 4.344855 |
| Huntington's disease | 4.54671 |
| Pathways in cancer | 3.67099 |

**Newly associated pathways via the complementary network**

Viral carcinogenesis, PI3K-Akt signaling pathway, NF-kappa B signaling pathway, Non-alcoholic fatty liver disease (NAFLD), Apoptosis, Hepatitis C, mTOR signaling pathway, MAPK signaling pathway, Jak-STAT signaling pathway, Insulin signaling pathway, Calcium signaling pathway, p53 signaling pathway, Basal transcription factors, Adipocytokine signaling pathway, Oxidative phosphorylation

**Visualization of the complementary network (green nodes = newly associated)**


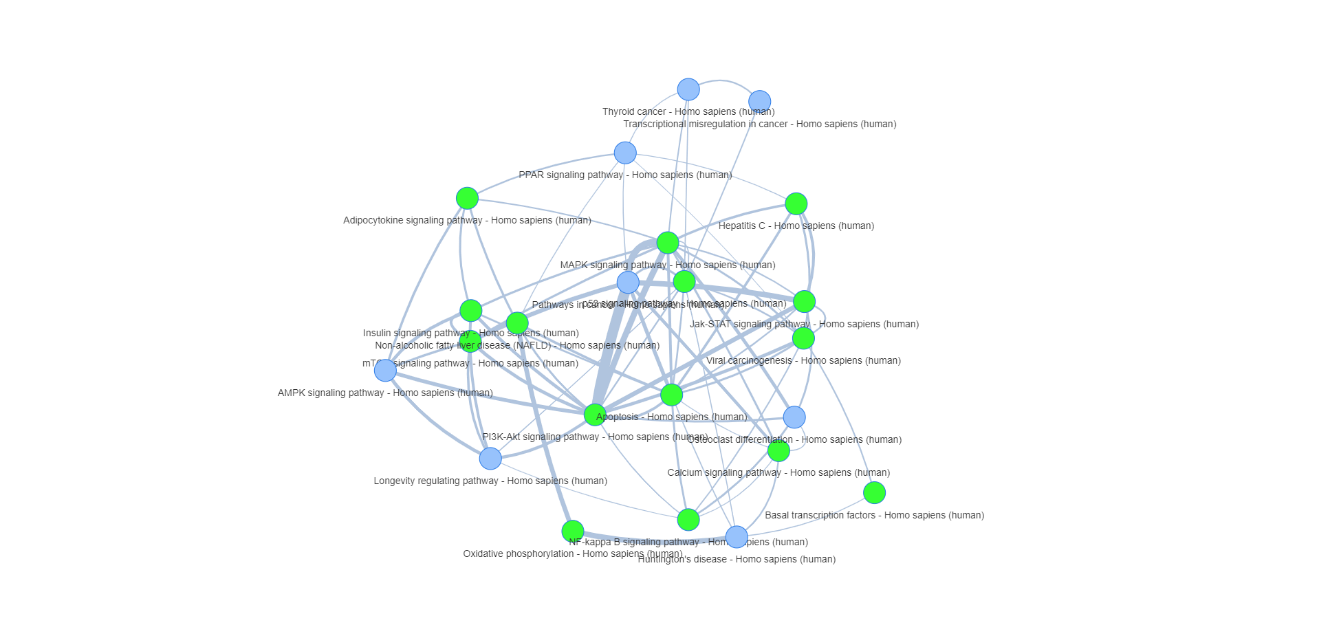


# Ulcerative Colitis

**Top 10 statistically significant pathways after enrichment**

| **Description** | **Combined Score** |
| --- | --- |
| SNARE interactions in vesicular transport | 8.144566 |
| VEGF signaling pathway | 7.391471 |
| B cell receptor signaling pathway | 6.845003 |
| Choline metabolism in cancer | 5.999651 |
| T cell receptor signaling pathway | 5.660978 |
| Axon guidance | 4.947552 |
| Osteoclast differentiation | 5.065285 |
| Natural killer cell mediated cytotoxicity | 4.910702 |
| Wnt signaling pathway | 4.849445 |
| Hepatitis B | 4.891771 |

**Newly associated pathways via the complementary network**

Kaposi's sarcoma-associated herpesvirus infection, Pathways in cancer, Proteoglycans in cancer, Hepatocellular carcinoma, Focal adhesion, MAPK signaling pathway, Parathyroid hormone synthesis, secretion and action, Endocytosis, TGF-beta signaling pathway, p53 signaling pathway, Ubiquitin mediated proteolysis

**Visualization of the complementary network (green nodes = newly associated)**


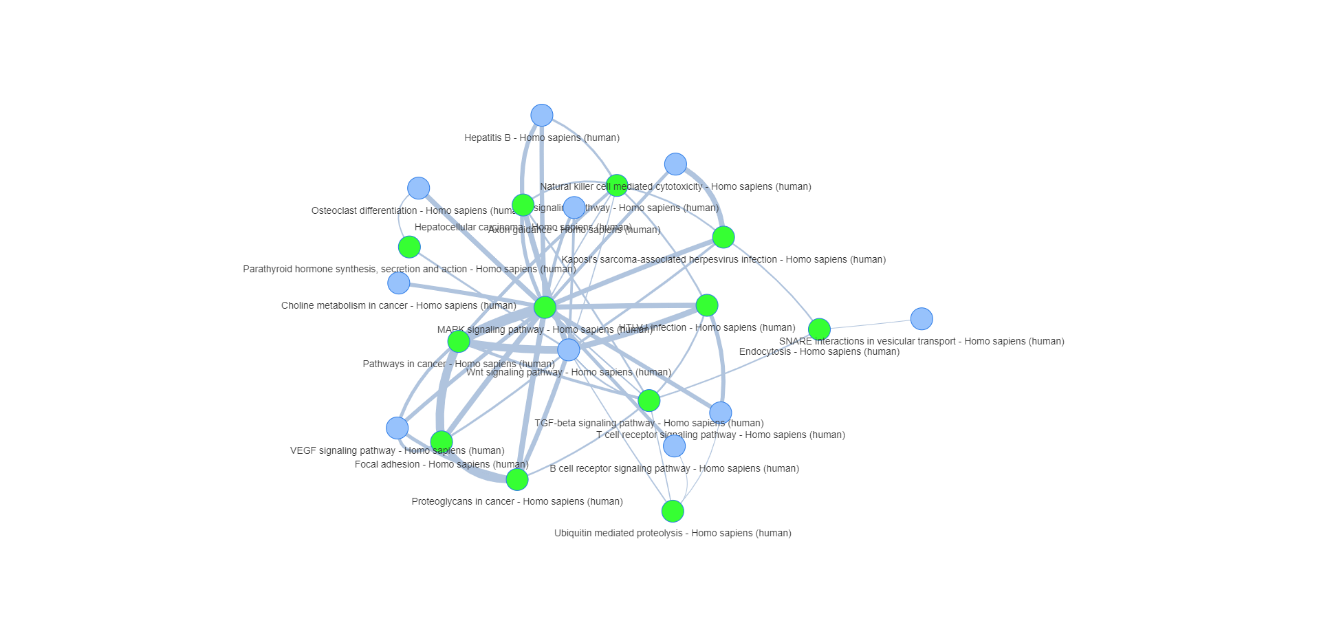


# UC E1 subphenotype

**Top 10 statistically significant pathways after enrichment**

| **Description** | **Combined Score** |
| --- | --- |
| Osteoclast differentiation | 13.097704 |
| RIG-I-like receptor signaling pathway | 6.320374 |
| Leishmaniasis | 6.659327 |
| Leukocyte transendothelial migration | 5.284945 |
| Phagosome | 4.740785 |

**Newly associated pathways via the complementary network**

PI3K-Akt signaling pathway, Epstein-Barr virus infection, Influenza A, NF-kappa B signaling pathway, Toll-like receptor signaling pathway, HTLV-I infection, Herpes simplex infection, Measles, Apoptosis, Hepatitis C, Systemic lupus erythematosus, Hepatitis B, MAPK signaling pathway, Jak-STAT signaling pathway, Rheumatoid arthritis, Endocytosis, Calcium signaling pathway, Cell adhesion molecules (CAMs), Fc gamma R-mediated phagocytosis, Intestinal immune network for IgA production, Complement and coagulation cascades, Regulation of actin cytoskeleton, Antigen processing and presentation, Ubiquitin mediated proteolysis

**Visualization of the complementary network (green nodes = newly associated)**

**
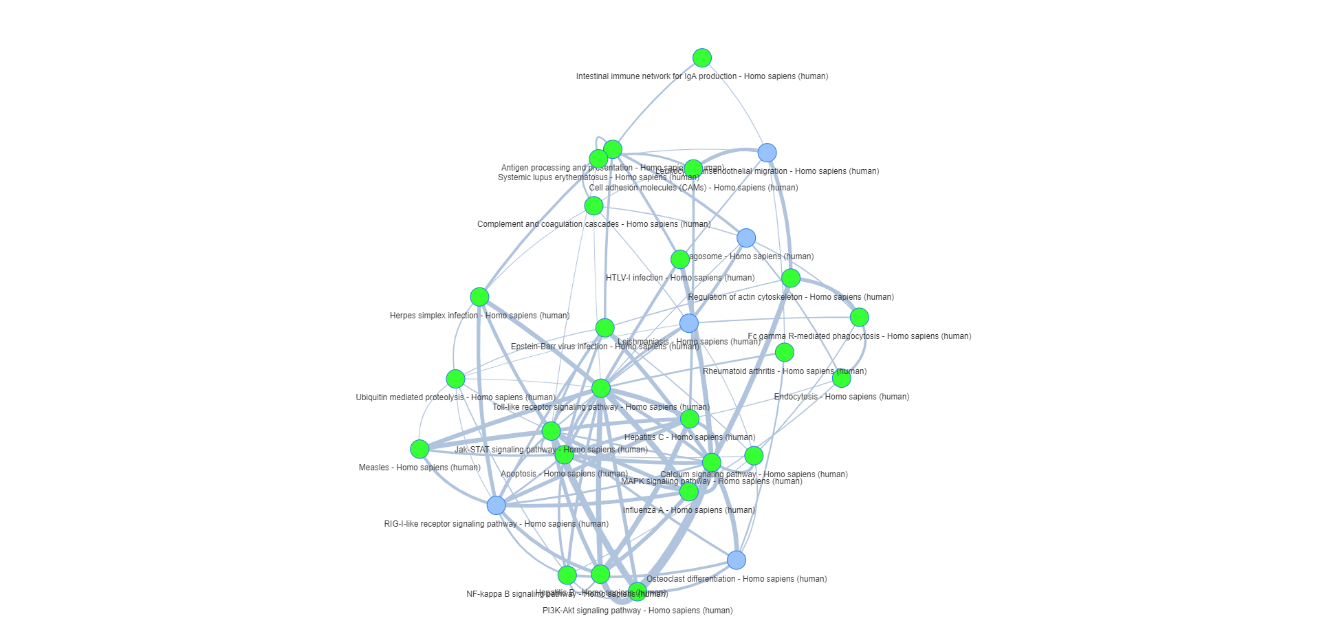
**

# UC E3 subphenotype

**Top 10 statistically significant pathways after enrichment**

| **Description** | **Combined Score** |
| --- | --- |
| SNARE interactions in vesicular transport | 7.54655 |
| VEGF signaling pathway | 6.768247 |
| B cell receptor signaling pathway | 6.240694 |
| Choline metabolism in cancer | 5.421347 |
| T cell receptor signaling pathway | 5.110807 |
| Axon guidance | 4.438165 |
| Osteoclast differentiation | 4.537768 |
| Natural killer cell mediated cytotoxicity | 4.395839 |
| Wnt signaling pathway | 4.333201 |
| Hepatitis B | 4.366603 |

**Newly associated pathways via the complementary network**

Kaposi's sarcoma-associated herpesvirus infection, Pathways in cancer, Proteoglycans in cancer, Hepatocellular carcinoma, Focal adhesion, MAPK signaling pathway, Parathyroid hormone synthesis, secretion and action, Endocytosis, TGF-beta signaling pathway, p53 signaling pathway, Ubiquitin mediated proteolysis

**Visualization of the complementary network (green nodes = newly associated)**

**
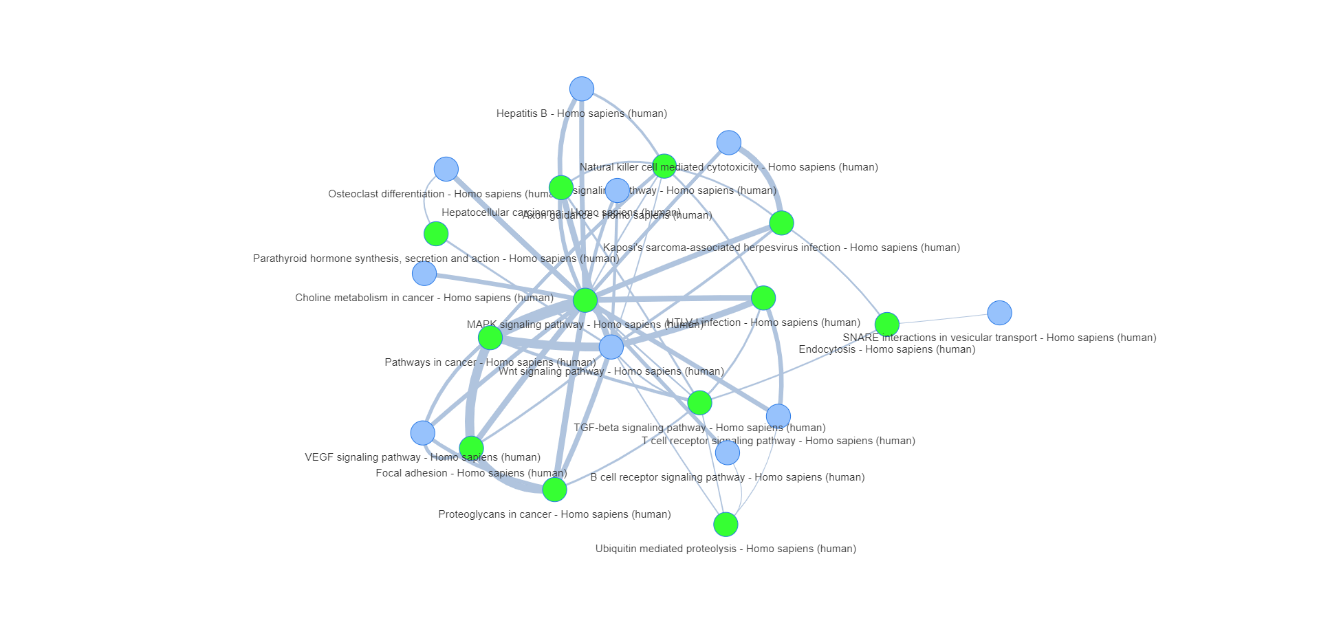
**
